# Assessment of Erosion Characteristics in Purple and Yellow Soils Using Simulated Rainfall Experiments

**DOI:** 10.3390/ijerph19010357

**Published:** 2021-12-30

**Authors:** Banglin Luo, Zhen Han, Jing Yang, Qing Wang

**Affiliations:** College of Forestry, Guizhou University, Guiyang 550025, China; luobl2021@163.com (B.L.); jyang13@gzu.edu.cn (J.Y.); hellowangqing@163.com (Q.W.)

**Keywords:** erosion, particle size distribution, sediment concentration, slope factor

## Abstract

Soil erosion of sloped lands is one of the important sources of substantive sediments in watersheds. In order to investigate erosion characteristics of sloped lands during rainfall events in the Three Gorges Reservoir Area, erosion processes of purple and yellow soils under different slope gradients and rainfall intensities were studied by using a rainfall simulator. The results showed that the sediment concentration in runoff was closely correlated with rainfall intensity. The sediment concentration in runoff gradually rose to a peak with time, and then gradually declined and approach a steady rate during simulation rainfall events. The particle size distribution of surface soils before the rainfall was different from that after the rainfall. Soil erosion mainly resulted in the loss of fine particles of surface soil through runoff, and the fine particles of soil were enriched in sediments. Soil erosion rates were gradually increased with the slope gradient when the slope gradient was less than 10°, and significantly increased when the slope gradient was more than 10°. The slope factor of yellow soil could be fitted well to that calculated by the formula of Universal Soil Loss Equation (USLE). The trend of the slope factor of purple soil was similar to that of the slope factor that was derived from USLE. Therefore, the effect of slope gradients on soil erosion need to be further researched when USLE was applied to predict erosion in purple soil area.

## 1. Introduction

Soil erosion is a major environmental concern in agricultural areas, especially in hilly areas and sloped farmlands [1], often leading to severe land degradation and unexpected water eutrophication [2]. Worldwide, there are around 800 million individuals who depend directly on steep lands with slope gradients of >20% for sustenance [3]. The soil erosion of sloped farmland is a tremendous threat to the sustainable development of agriculture in mountainous and hilly regions [4]. On the one hand, soil erosion in sloped farmland is one of the important sources of substantive sediments in rivers. On the other hand, the serious erosion process in sloped farmland makes the soil’s depth thin, nutrient deficient, as well as crop yield decrease. Soil erosion is subject to multifarious factors such as rainfall intensity, soil erodibility, slope gradient, slope length, and cover-degree of vegetation [5,6]. 

Rainfall intensity, which via controlling the hydrologic response with higher rainfall intensity generates higher runoff peak, is one dominant factor of the rainfall-runoff and soil erosion processes [6]. The slope gradient of the soil surface is a crucial factor that would remarkably influence soil erosion. The slope gradient controls the hydrologic response and has been extensively studied via numerical simulation, experiments, and analytical solutions, and is still the hot topic of soil erosion research [5,6]. Generally, increased slope gradient is associated with increased sediment transport [7]. Some studies reported that the erosion rate of cultivated land is mainly affected by water content, while that of uncultivated land is mainly affected by slope gradient [8]. The assessment of soil loss and the influence factors have been carried out using estimates such as the Universal Soil Loss Equation (USLE, as a simplest mathematical model proposed by, is an empirical quantitative model designed for the evaluation of the annual soil loss [9]), Revised Universal Soil Loss Equation (RUSLE), and Water Erosion Prediction Project (WEPP, is a daily simulation model keeping account of the hydrologic status of the land and biomass with erosion predictions being generated when runoff is predicted to occur [10]). As the most complicated physical model by far, WEPP is a continuous simulation based on gully erosion and inter-gully erosion process, and sediment transport mechanism. Compared with the USLE, the biggest advantage of WEPP is that it can estimate the temporal and spatial distribution of slope soil erosion [11]. However, for the soil erosion in mountainous areas, both USLE and WEPP need to be further improved due to the complex terrain conditions and numerous influencing factors. 

Soil erosion processes include particle detachment, transportation, and deposition. In the early stage of rainfall erosion, raindrops directly fall on soil surfaces and cause soil particles to disperse, separate, and move. Thus, rain splash erosion occurs and then stimulates water erosion [12,13,14]. In addition to runoff and soil loss, the size of sediment particles is a significant factor influencing detachment and transport [15,16]. Hence, sediment particle size distribution has been extensively investigated [17,18,19]. It is demonstrated that soil clay fraction and erosion index exhibit a negative linear relationship, and sand content and erosion index show a positive curve [20]. Stable aggregates can reduce particle dispersion after either natural or artificial rainfall. <0.02 mm sediment aggregates and <0.002 mm clay are enriched during soil loss in purple soil [12]. The loss of sediment content of fine particles decreases with time, and sediment clay exhibits enriching properties. 

Although there has been much research on erosion characteristics, the research in the Three Gorges Reservoir Area is still insufficient due to the particularity of the environment. The area and rate of soil and water loss in the Three Gorges Reservoir Area have increased for the past 40 years because of irrational land use. The quantity of slope land erosion accounts for 60% of the total soil erosion in the Three Gorges Reservoir Area. Soil erosion has been a serious environmental and production problem in this area. Purple and yellow soils (the description of these soils is available in Section 2.1) are the major soils in the Three Gorges Reservoir Area [21]. In this study, runoff and sediment processes, the particle size distribution of sediment, and erosion estimation of purple and yellow soils were studied using a rainfall simulator. The aim of this study was to identify how the soil loss response to the rainfall intensity and slope gradient, clarify the particle size distribution in surface soils before and after rainfall and eroded sediments, and discuss the applicability of the USLE and WEPP in the study area. 

## 2. Materials and Methods

### 2.1. Study Area

**Figure 1:**
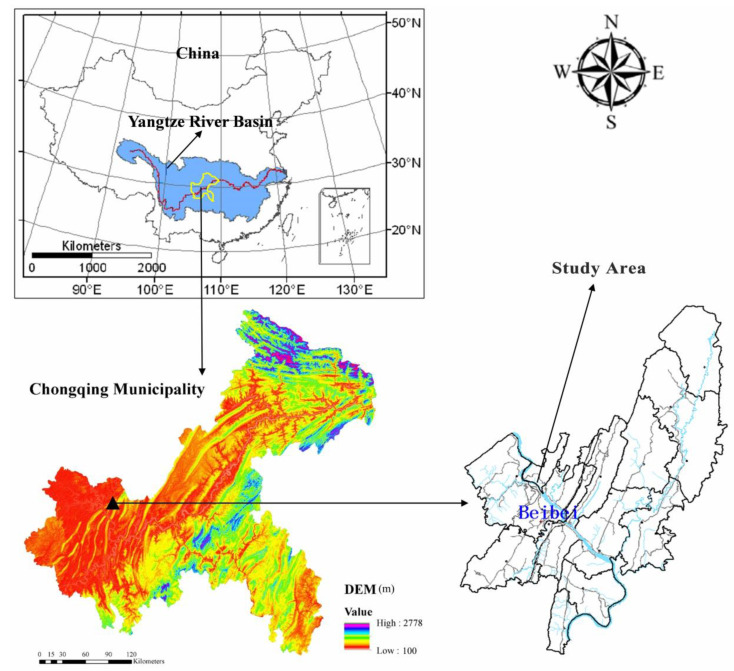
The study area.

The study area is located in Beibei, Chongqing, China (106°26′ E, 30°26′ N) at an altitude of 230 m (Figure 1). The area has a subtropical, humid climate with a mean annual temperature of 18.2 °C and a mean annual precipitation of 1105 mm, with most of the rainfall concentrated in the summer, accounting for 70% of the annual rainfall. The average annual amount of sunshine is 1277 h, and the mean annual frost-free period is 334 d.

### 2.2. Soil Testing and Analysis

The test soil used in this study was collected from the study area. The purple soil (Purpli-Udic Cambisols), developed from gray-brown purple sandy mudstone of Shaximiao Formation of Jurassic system. The yellow soil (Alliti-Perudic Ferrosols), formed from the limestone of the Trias system. According to the methods of soil and plant analysis [22], the pH was measured using a pH meter. The organic matter (OM) content of the soil was tested by oxidation with potassium dichromate (K_2_Cr_2_O_7_). Total nitrogen (TN) of the soil was determined by the Kjeldahl method. The available nitrogen (AN) of the soil was determined via 1.0 mol L^−1^ NaOH extraction and distilling. Total phosphorus (TP) of the soil was measured by colorimetry after digestion with HClO_4_ + H_2_SO_4_, and the available phosphorus (AP) was measured by colorimetry after extraction with 0.5 mol L^−1^ NaHCO_3_ (pH = 8.5). The total potassium (TK) of the soil and available potassium (AK) of the soil was measured by a flame photometer using the NaOH fusion method and extraction with NH_4_OAc, respectively. The physical and chemical properties of the test soils were shown in Table 1. 

### 2.3. Experimental Facilities and Rainfall Simulation

**Figure 2:**
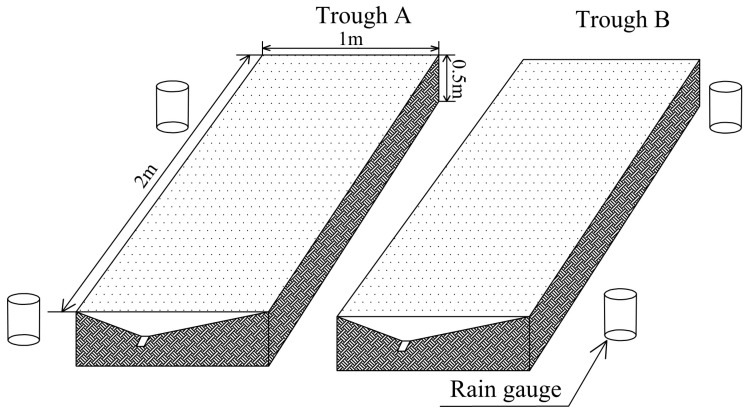
Experimental setup of rainfall simulation experiment with two parallel 2 m × 1 m × 0.5 m troughs.

Rainfall simulation experiments were conducted at the soil and water conservation experimental hall at Southwest University. Tap water (electrical conductivity 0.7 dS m^−1^) was used in all of the experiments. The simulated rainfall used was produced by a down spray rainfall simulator with a rainfall height of 1.5 m. The simulator consists of an in-line filter to prevent nozzle clogging, a pressure regulator, a flow meter, and a pressure gauge to control flow [23]. The dynamics of rainfall were monitored using four electronic rain gauges placed at the edges of the troughs. Experiments were conducted in two parallel 2 m × 1 m × 0.5 m (long × wide × deep) troughs (called A and B), which were shown in Figure 2.

The soils were sieved with a 10 mm opening sieve before the soils were packed in the experimental flume. To ensure better water permeability, a 10 cm deep sand layer was loaded in the bottom of the troughs and covered with gauze. Then, the troughs were filled with a 5 cm to 30 cm-deep layer of till to imitate the tillage layer. Each layer was bristled with a brush to minimize boundary effects. Soil bulk density was 1.3 g cm^−3^, and the moisture content of the soil is controlled to be about 18%. Once the troughs were prepared, Slope gradients were controlled 5°, 10°, 15°, 20°, 25°, and 30° artificially. Moreover, the rainfall intensities were calibrated to 1.8, 3.0, 4.0 mm min^−1^ and measured three times to ensure that the error was less than 5%. When the rainfall intensity was sufficiently calibrated, the plastic sheeting was removed. During rainfall simulations, the initiation time of runoff was recorded. Runoff and sediment were collected at 5-min intervals, and 150 mL samples of runoff that contained sediment were collected in a beaker at 5-min intervals.

### 2.4. Index Determination

After the tests were completed, the sediments were oven-dried at 105 °C for >24 h to calculate the sediment yields. Additional 150-mL sediment-laden water samples were collected for the measurement of the particle distribution of the sediments. The particle size distribution of the sediments in the water samples were obtained using a Malvern Mastersizer 2000 laser diffraction instrument. The sediment samples were mixed until homogenous. A sample of the sediment mixture (1–2 mL) was added to a beaker containing 800 mL of distilled water and left for 2 min to obtain the particle size distribution of the sediments.

## 3. Results and Discussion

### 3.1. Soil Loss Response to the Rainfall Intensity and Slope Gradient

**Figure 3:**
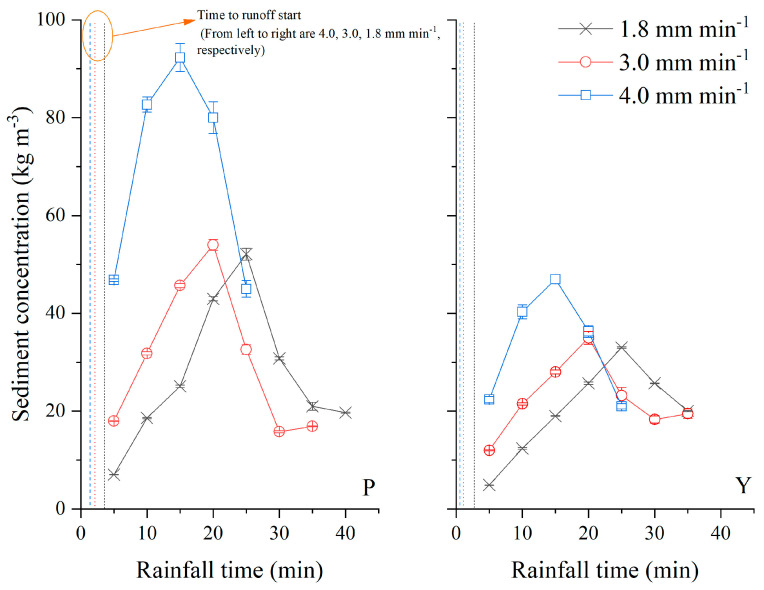
Sediment concentration in runoff with rainfall time under different rainfall intensities at slope 15° (P represents purple soil, Y represents yellow soil).

As shown in Figure 3, the yellow soil produced runoff earlier than the purple soil. Due to the loose structure and coarse particles, the purple soil has a higher infiltration capability, so infiltration gave first at the initial stage of rainfall. After the runoff started, sediment concentration in runoff of purple and yellow soils gradually rose up and reached to peak, then gradually decreased and approach a steady rate in all rainfall intensities. The concentration reduced with rainfall time as a consequence of the exhaustion of loose surface material. The properties of peaks of sediment concentration in runoff water were highly related to rainfall intensity. The stronger the rain intensity was, the earlier the appearance time of peak was, and the higher the peak value was. In this study, at 15° slope gradient, peak values of sediment concentration in runoff of purple and yellow soils under rainfall intensities of 4.0, 3.0, and 1.8 mm min^−1^ were 92.3, 45.7, and 43.0, and 47.0, 35.0, and 33.0 kg m^−3^, separately (Figure 3). The peak time in purple and yellow soils under rainfall intensities of 4.0, 3.0, and 1.8 mm min^−1^ appeared at 15, 20, and 25 min after the runoff started, respectively. 

For the same slope gradient and rain intensity, the peak value of purple soil was 1.42 times that of yellow soil at low rain intensity of 1.8 mm min^−1^. The difference between the peak value of purple and yellow soils increased with the rain intensity, and the peak value of purple soil was 1.96 times that of yellow soil when the rainfall intensity was 4.0 mm min^−1^. However, the change of sediment concentration in runoff had the same trend during the rainfall events for both soils, as shown in Figure 3. The sediment concentration in runoff of purple and yellow soils was approximated to 20 kg m^−3^ at the end of each rainfall event, indicating the dynamic equilibrium between runoff and anti-erodibility of surface soil. At the same slope gradient, the discrepancies of sediment concentration in runoff of purple soil under various rainfall intensities were greater than those of yellow soil. This implied that the structure of yellow soil could be more stable than that of purple soil.

**Figure 4:**
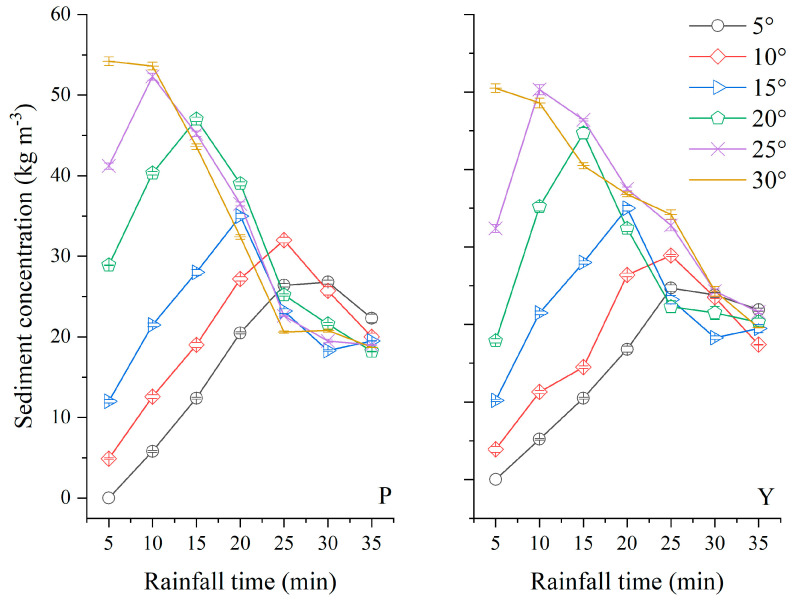
Sediment concentration in runoff with rainfall time under different slope gradients in rainfall intensity of 3.0 mm min^−1^ (P represents purple soil, Y represents yellow soil).

Figure 4 showed changes of sediment concentration in runoff during the rainfall event rainfall intensity of 3.0 mm min^−1^ under different slope gradients. The average sediment concentration in runoff of purple and yellow soils increased with slope gradient. With the increase of slope gradient, the gravitational potential energy of runoff increases continuously. And the capacity of sediment transport increases accordingly, which leads to the increasing sediment concentration in runoff. In Figure 4, the average sediment concentration in runoff of purple soil at 30° slope gradient was 2.14 times like that at 5° slope gradient and 1.28 times as that at 15° slope gradient when the rain intensity was 3.0 mm min^−1^, and 2.51 times and 1.64 times for yellow soil, respectively. The peak value of sediment concentration in runoff appeared earlier and was higher with the steepness of the slope. The peak time of purple and yellow soils at 30° slope gradient appears at 5 min after the runoff started, while 30 and 25 min at 5° slope gradient, separately. The peak value of sediment concentration in runoff of purple soil at 30° slope gradient was highest in the experiment and was 1.97 times as that in yellow soil under the same condition. As a while, the sediment concentration in runoff of purple soil was much higher than that in runoff of yellow soil. The anti-erodibility of yellow soil is stronger than that of purple soil.

### 3.2. Particle Size Distribution in Surface Soils before and after Rainfall and in Eroded Sediments

The dispersion and initiation of the soil particles on the slope surface are the beginning of erosion. The detached soil particles, especially clay particles, are important carriers of soil nutrients and pollutants [24], which have an important impact on the agricultural ecological environment. Therefore, it is necessary to examine the distribution and sorting characteristics of the eroded sediment particles. The particle size distribution of surface soils before the experiment somewhat differed from that after the experiment, as shown in Table 2. The contents of gravel (>2.0 mm), coarse- sand (2.0–0.2 mm), and fine-sand (0.02–2 mm) in the purple and yellow soils before experiments were slightly increased, and the contents of silt (0.002–0.02 mm) and clay (<0.002 mm) were decreased in comparison with those after experiments. The results from the discrepancy of the particle size distribution in the surface soil before and after rainfall event were that silt (0.002–0.02 mm) and clay particle (<0.002 mm) were markedly enriched in sediment. The gravels (>2.0 mm) were not generally found in the sediments. The particle size distribution of sediment was mainly characterized by <0.02 mm particles, which approximated 80% of sediment. The contents of silt (0.002–0.02 mm) and clay particle (<0.002 mm) of sediments were 29.4% and 23.6%, 61.9%, and 8.4% higher than those of surface soil in the purple and yellow soils, respectively. The <0.02 mm soil particles, especially clay particles (<0.002 mm), greatly exist in soil organic-mineral particles, which contain nutrient cations and anion. The finer the sediment particles are, the larger the specific surface area per unit volume of sediment particles, which are an important carrier of soil nutrients and pollutants. At this time, the physical and chemical action of the particle surface also plays a very important role in scouring, transportation, and deposition [12]. Consequently, soil erosion results in crop yield decrease in agricultures because mainly of the loss of fine particles of surface soil. 

**Figure 5:**
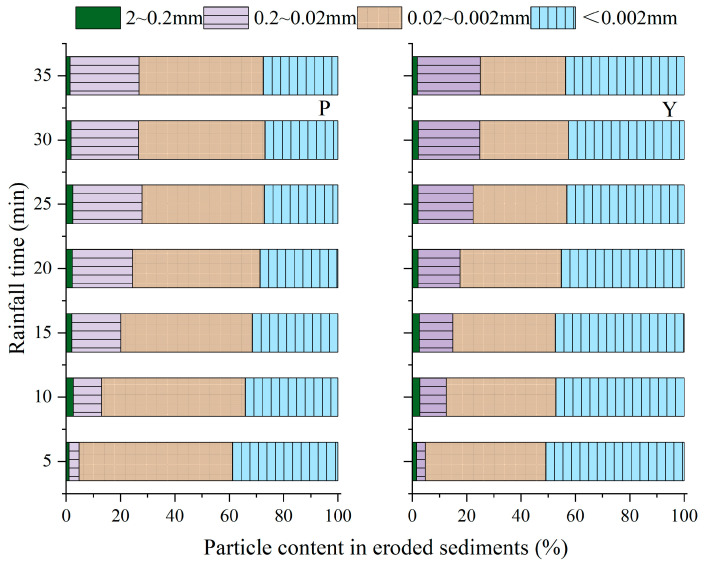
Particle content in eroded sediments with rainfall time at slope 15° and 3.0 mm min^−1^ rain intensity.

A time-dependent size distribution of the eroded sediments was observed during rainfall processes. The content of silt (0.002–0.02 mm) and clay (<0.002 mm) particle contents gradually declined, and then approached stability (Figure 5). While the contents of 0.02–0.2 mm particles in sediments of purple and yellow soils were significantly changed from 3.76% to 25.48% and from 3.24% to 23.13% during rainfall events, separately. Therefore, in the early stage of the runoff, the particles in sediments were fundamentally characterized by <0.02 mm particles, occupying 95% of sediments. By contraries, contents of 0.02–0.2 mm particles in sediments approximately account for 25% of sediments in the later stage of the runoff. This result was not consistent with the result obtained by Martínez-Mena et al. [25] using simulated rainfall in a semiarid Mediterranean area of SE Spain, in which coarse fractions decreased and fine fractions increased with rainfall time under rainfall intensities of 56.0 ± 2.4 mm h^−1^ and 31.4 ± 1.4 mm h^−1^. This distinction may have originated from the rainfall intensity used in the experiments. Higher rainfall intensity results in a higher runoff, which makes it possible to transport more coarse particles in runoff. The time to runoff will also affect the size distribution of the eroded sediment. The later runoff generation time indicates that the infiltration process is longer. In this process, the water flow will disperse the soil particles, causing the soil particles to disintegrate and produce more fine particles, thus increasing the content of fine particles in eroded sediment. On the contrary, if the runoff generation time is earlier, the soil does not have enough time to disintegrate. At this time, under the condition of heavy rainfall intensity, coarse particles will be eroded first. With the continuation of rainfall, the content of coarse particles will decrease, and the content of fine particles will increase. 

### 3.3. Relationship between Erosion Rate and Slope Gradient

The transport force (shear force of runoff) and compaction force working on the soil particles are significantly interrelated to the rainfall intensity and slope gradient [26]. The transport force along the surface of the soil was positively correlated with slope gradient, and the compaction force was a negative correlation with slope gradient. Transport force becomes stronger, and compaction fore become weaker with the increasing slope gradient. So, the soil particles of the steeper sloping field are easily washed and result in substantive soil erosion. 

Detachment is the dislodging of particles from the soil matrix by erosive agents. This occurs by several processes, predominant of which are the hydraulic forces of raindrop impact and surface runoff on the surface soil. The surface soil could not be detached by the transport force in a small slope gradient (<10°), therefore the discrepancy of soil erosion between purple and yellow soils was slight. But when the slope gradient was >10°, the surface soil was dispersed and the rill erosion occurred because the transport force was higher than the resistance to the shear force of surface soil. Accordingly, soil erosion of purple soil was evidently higher than that of yellow soil in steep slope gradient (>10°).

The prediction equations of soil erosion for purple soil and yellowed soil were set up by regression analysis of the data obtained from the experiments. The estimation equation of soil erosion for purple soil was followed as,

*E_r_* = 0.15 + 0.027*s* + 0.07*r* + 0.12*sr R*^2^ = 0.87 (1)

The estimation equation of erosion rate for yellow soil was regressed as,

*E_r_* = −0.35 + 0.021*s* + 0.059*r* + 0.078*sr R*^2^ = 0.81 (2)

where *E_r_* is erosion rate (kg m^−2^); *s* is slope gradient (°) and *r* is average rainfall intensity (mm min^−1^). 

### 3.4. Slope Factor

Slope gradient is a crucial factor working on soil erosion, and the effect can be evaluated by the slope factor. In the early version of the USLE, soil erosion was estimated as a power function of slope gradient. The power relationship of the USLE overestimates soil erosion rate by as much as two to three times and the relationship is better described as linear or less than linear [27]. In this paper, the slope factor was discussed with the following two formulas:

The slope factor calculation formula of USLE was defined as

(3) $$S=64.51\sin^{2}\alpha +4.56\sin\alpha +0.065$$

The slope factor calculation formula of WEPP was defined as

(4) S=2.19{1.05−0.85exp[(−4)sinα]}

where *α* is slope gradient (°), and *S* is slope factor, which means the ratio of soil erosion mass between actual slope gradient and normative slope gradient (sin *α* = 9%, *α* = 5.16°).

**Figure 6:**
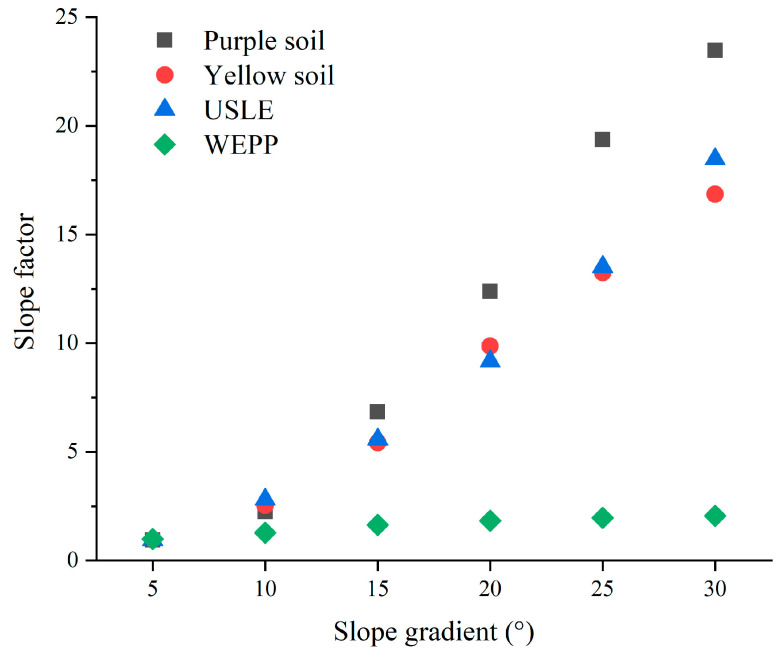
Comparison of calculated and measured values of slope factors for two kinds of soils.

Equations (3) and (4) are the slope factor formula of USLE and WEPP, respectively. The calculated and observed values of the slope factor for purple and yellow soils were shown in Figure 6. Both slope factors of purple and yellow soils increased with the slope gradient but were not well fitted to values calculated by the slope factor formula of WEPP. However, both slope factors of purple and yellow soils could somewhat be fitted to the slope factor calculation formula of USLE. When the slope gradient was less than 10°, the slope factors of purple and yellow soils could be well fitted to the slope factor calculation formula of USLE. When slope gradient was more than 10°, the slope factors of purple soil were obviously higher than those calculated from the slope factor formula of USLE, and there was not too much distinction between the slope factors of yellow soil and the slope factors calculated from the slope factor formula of USLE. Therefore, as the slope factor was concerned, USLE could be used to estimate soil erosion of yellow soil. The research of Miao [11] showed that WEPP was not ideal for predicting the rainfall process with less sediment yield. The results of this study also proved this point, but the specific reasons need further research. With the increasing slope gradient, the erosion process became more intense, and the conversion of erosion energy became more frequent. Under the condition of increasing rainfall intensity, this situation would be more obvious. The erosion process was not only accompanied by surface erosion, gully erosion, and small-scale collapse, which also affected the accuracy of the model prediction. Therefore, considering the influence factors of the soil erosion process under complex conditions is still the focus of model prediction development. 

## 4. Conclusions

The erosion characteristics of purple and yellow soils were studied using simulated rainfall experiments. The results showed that the sediment concentration in runoff was closely correlated to rainfall intensity and slope gradient. The properties of peaks of sediment concentrations in the runoff were related to rainfall intensity, the stronger the rainfall intensity was, the earlier the appearance time of the peak was, and the higher the peak value was. In the same way, the steeper the slope was, the earlier the appearance of peak was, and the higher the peak value was. In the same rainfall intensity, the steeper the slope was, the higher the sediment concentration of runoff was in the corresponding period. The average sediment concentrations in runoff of purple and yellow soils were significantly increased with slope gradient. The particle size distribution of sediments was mainly characterized by <0.02 mm soil particles, which approximate 80% in sediments. Consequently, soil erosion results in crop yield decrease in agricultures because mainly of the loss of fine particles of surface soil. Both slope factors of purple and yellow soils were not well fitted to values calculated by the slope factor formula of WEPP. As the slope factor was concerned, the slope factor of the yellow soil could be well described with the slope factor calculation formulas of USLE. The parameters of USLE must be modified when USLE was applied to predict soil erosion of purple soil.

## Figures and Tables

**Table 1:** The properties of the test soils in the experiment *.

Soil Type	pH	OM (g kg^−1^)	TN (g kg^−1^)	AN (mg kg^−1^)	TP (g kg^−1^)	AP (mg kg^−1^)	TK (g kg^−1^)	AK (mg kg^−1^)
P	6.9	17	0.96	111	0.57	5.1	28.51	65.2
Y	7.5	24.9	1.5	123	0.76	7.5	19.14	87.1

* P—purple soil, Y—yellow soil, OM—organic matter, TN—Total nitrogen, AN—available nitrogen, TP—Total phosphorus, AP—available phosphate, TK—total potassium, AK—available potassium.

**Table 2:** Particle size distribution in surface soils before and after rainfall and in eroded sediments at slope 15° and 3.0 mm min^−1^ rainfall intensity.

Soil Type	Items	>2 mm (%)	0.2–2 mm (%)	0.02–0.2 mm (%)	0.002–0.02 mm (%)	<0.002 mm (%)
P	Before rainfall	13.03	2.19	30.36	32.81	21.60
	After rainfall	12.87	3.19	34.44	28.63	20.87
	Eroded sediment		1.95	18.56	48.82	30.67
Y	Before rainfall	4.32	8.75	27.41	27.04	32.48
	After rainfall	5.85	9.66	29.35	24.87	30.28
	Eroded sediment		2.16	15.28	45.76	36.80

## Data Availability

Not applicable.
